# Types and Outcomes of Arrhythmias in a Cardiac Care Unit in Western Kenya: A Prospective Study

**DOI:** 10.5334/gh.1261

**Published:** 2023-09-21

**Authors:** Joan Kiyeng, Constantine Akwanalo, Wilson Sugut, Felix Barasa, Ann Mwangi, Benson Njuguna, Abraham Siika, Rajesh Vedanthan

**Affiliations:** 1 Department of Cardiology, Moi Teaching and Referral Hospital, Eldoret, KE; 2 Department of Medicine, Moi University School of Medicine, Eldoret, KE; 3 Department of Math, Physics and Computing, Moi University, Eldoret, KE; 4 Department of Clinical Pharmacy & Practice, Moi Teaching and Referral Hospital, KE; 5 Department of Population Health and Department of Medicine, NYU Grossman School of Medicine, New York, USA

**Keywords:** Arrhythmias, Atrial Fibrillation, Cardiac Care Unit

## Abstract

**Background:**

Sustained arrhythmias are frequently encountered in cardiac care units (CCU), but their types and outcomes in Africa are unknown. Studies from high-income countries suggest arrhythmias are associated with worse outcomes.

**Objectives:**

To determine the types and proportion of cardiac arrhythmias among patients admitted to the CCU at Moi Teaching and Referral Hospital (MTRH), and to compare 30-day outcomes between patients with and without arrhythmias at the time of CCU admission.

**Methods:**

We conducted a prospective study of a cohort of all patients admitted to MTRH-CCU between March and December 2021. They were stratified on the presence or absence of arrhythmia at the time of CCU admission, irrespective of whether it was the primary indication for CCU care or not. Clinical characteristics were collected using a structured questionnaire. Participants were followed up for 30 days. The primary outcome of interest was 30-day all-cause mortality. Secondary outcomes were 30-day all-cause readmission and length of hospital stay. The 30-day outcomes were compared between the patients with and without arrhythmia, with a *p* value < 0.05 being considered statistically significant.

**Results:**

We enrolled 160 participants. The median age was 46 years (IQR 31, 68), and 95 (59.4%) were female. Seventy (43.8%) had a diagnosis of arrhythmia at admission, of whom 62 (88.6%) had supraventricular tachyarrhythmias, five (7.1%) had ventricular tachyarrhythmias, and three (4.3%) had bradyarrhythmia. Atrial fibrillation was the most common supraventricular tachyarrhythmia (82.3%). There was no statistically significant difference in the primary outcome of 30-day mortality between those who had arrhythmia at admission versus those without: 32.9% versus 30.0%, respectively (*p* = 0.64).

**Conclusion:**

Supraventricular tachyarrhythmias were common in critically hospitalized cardiac patients in Western Kenya, with atrial fibrillation being the most common. Thirty-day all-cause mortality did not differ significantly between the group admitted with a diagnosis of arrhythmia and those without.

## Introduction

Arrhythmias are common among critically ill cardiac patients and are associated with increased morbidity and mortality [1,2,3]. They may be the primary reason for admission or may occur among admitted critically ill patients [4]. In patients with structural heart disease, arrhythmias are not only a marker but also a predictor of both severity and adverse outcomes [5]. However, most available data are from high-income countries (HIC).

Data on arrhythmias in Africa are limited [6,7,8], however, the few available studies in outpatient settings show a wide discrepancy in the diagnosis and care of arrhythmias in comparison with HICs, which contributes to differences in both the types and outcomes of arrythmias that may be seen in this setting [9,10]. Atrial fibrillation (AF) is the most common type of arrhythmia worldwide among both the general population and the critically ill [11,12,13]. Notably, data from outpatient settings in Africa reveal inadequate care of patients with AF in comparison to the other continents [14,15,16]. Pacing, which is important for survival among patients with brady-arrhythmias is not widely available [17,18,19]. Further, in centers where pacing is available, the pacing devices are costly, and this is compounded by a scarcity of human resources [20]. In addition, there is poor response and inadequate reporting on sudden cardiac death (SCD) that is thought to occur after a ventricular arrhythmia [21,22,23,24]. There is, however, a need for data on the burden of other arrythmias in the inpatient setting and their impact on clinical outcomes in our African setting.

Whereas previous studies done in Africa focused on specific arrhythmias in an outpatient set up [16,25,26], this prospective study looked at all the types of arrhythmias and the 30-day outcomes in a cardiac care unit.

## Methods

### Study population and design

This was a prospective observational cohort study that was conducted in the CCU at MTRH, which is the only tertiary hospital in Western Kenya and the second largest referral Hospital in Kenya. After enrollment, the participants were followed up for 30 days or until death, whichever occurred first.

### Study procedure

After receiving approval from the Institutional Research and Ethics Committee of Moi University School of Medicine and MTRH (approval no: 0003766), all adult patients admitted to the CCU during the study period and having provided written informed consent, were consecutively recruited until a target sample size of 160 participants was achieved. We excluded patients who were unable/declined to consent, those who had undergone cardiopulmonary resuscitation within the prior 24 hours and those who were admitted for elective procedures. Each participant’s admission 12-lead ECG tracing was interpreted by the admitting cardiology fellow and confirmed by an attending cardiologist (CA, WS and FB). The participants were then stratified on the presence or absence of sustained arrhythmia irrespective of whether it was the primary indication for CCU care or not. Sinus tachycardia or asymptomatic sinus bradycardia of more than 40 beats/minute, premature supraventricular and ventricular contractions were excluded from arrhythmia diagnosis. Other variables that were collected included: demographics, clinical characteristics, admission diagnosis as per primary clinician, cardiac structure and function based on echocardiography results, clinical management, and laboratory findings.

The arrhythmias were classified broadly into ventricular tachyarrhythmias, supraventricular tachyarrhythmias, and bradyarrhythmia. Hemodynamic stability was dichotomized at admission. Arrhythmias were managed as per established guidelines or at the discretion of the attending cardiologist. The primary outcome was 30-day all-cause mortality, and the secondary outcomes were length of hospital stay and all cause readmission within 30 days after enrollment.

Participants were reviewed daily while in the hospital and a follow-up phone call was made at least weekly after discharge until death or up to 30 days after the recruitment, whichever occurred first. The length of hospital stay was defined as the entire hospital stay, and therefore included any time spent in the general medical wards. An ECG was obtained on discharge to establish if there was resolution of the arrhythmia. In case of a readmission within 30 days, a copy of the discharge summary was obtained and the reason for readmission was documented. For any reported mortality, a copy of the death certificate was obtained; if not possible, we sought confirmation of death from the area chief or sub-chief.

### Data analysis

Data was entered into a Red Cap database and analyzed using STATA version 15 and RGUI version 4.1.1. Descriptive statistics such as frequencies and proportions were used for categorical variables while means and medians and associated standard deviation and interquartile range were used for continuous variables. Cross-tabulation tables were used to describe patients’ profiles and outcomes by arrhythmia status at admission. Survival analysis was used to model mortality and presented using Kaplan-Meier curves. We also conducted multivariate analysis to assess factors associated with 30-day mortality.

## Results

**Figure 1:**
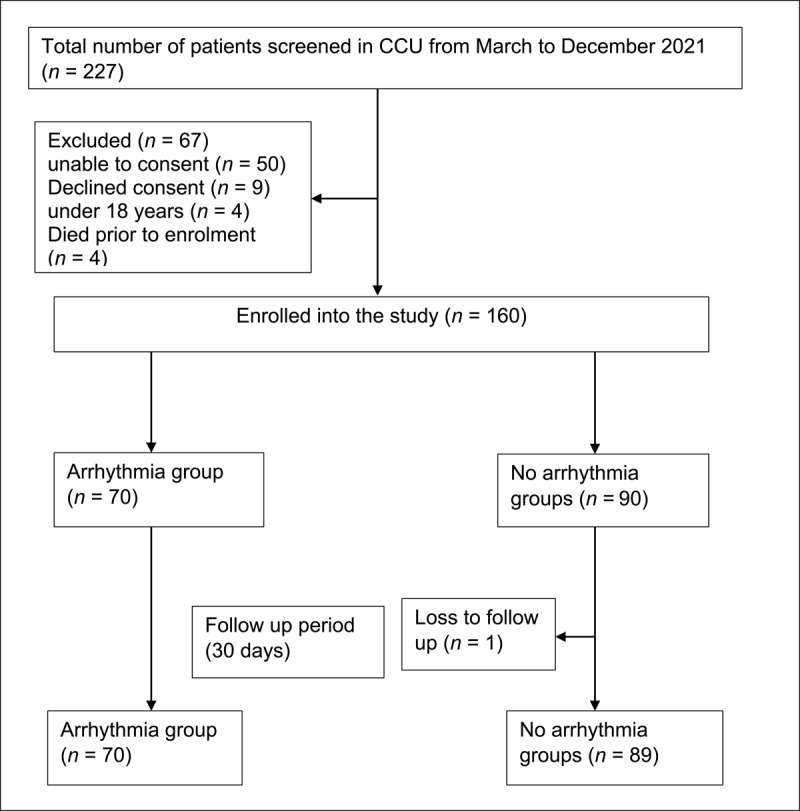
Flow diagram showing study recruitment schema.

A total of 227 patients admitted to MTRH-CCU between March and December 2021 were screened, and 160 patients were enrolled consecutively into the study. A total of 70 (43.7%) had a diagnosis of arrhythmia at admission. All patients were followed up for 30 days (Figure 1).

The median age of the patients was 46 (IQR = 31, 68). Most of the participants were female (59.4%), with a lower proportion of males in the arrhythmia group (31.4%) compared to the no arrhythmia group (47.8%) (*p* value = 0.04). A significantly high proportion of patients with arrhythmia had a history of known rheumatic heart disease (RHD) compared to those without arrhythmia [34.3% versus 3.3%, *p* value < 0.001]. The patients’ characteristics are presented in Table 1. At admission, RHD was the most common underlying structural heart disease in the arrhythmia arm (50%) while cor pulmonale and uncharacterized dilated cardiomyopathy were frequent in the no arrhythmia arm (Table 2).

**Table 1:** Patients’ characteristics.


PARTICIPANTS’ BASELINE CHARACTERISTICS	ALL	ARRHYTHMIA		*P* VALUE
	
	*N* = 160	YES (*N* = 70)	NO (*N* = 90)	

Median age in years (IQR)	46 (31, 68)	46 (29, 70)	46 (31, 63)	0.873

Female gender—*n* (%)	95 (59.4)	48 (68.6)	47 (52.2)	0.037

Heart failure at admission—*n* (%)	137 (85.6)	62 (88.6)	75 (83.3)	0.349

**NYHA Class**				0.195

I	12 (8.8)	3 (4.8)	9 (12.0)	

II	34 (24.8)	13 (21.0)	21 (28.0)	

III	73 (53.3)	35 (56.5)	38 (50.7)	

IV	18 (13.1)	11 (17.7)	7 (9.3)	

**Medical history—*n* (%)**				

Heart failure	80 (50)	41 (58.6)	39 (43.3)	0.056

Hypertension	28 (17.5)	11 (15.7)	17 (18.9)	0.6

Rheumatic heart disease	27 (16.9)	24 (34.3)	3 (3.3)	<0.001

Arrhythmia	18 (11.2)	18 (25.7)	0 (0.0)	<0.001

Diabetes mellitus	8 (5)	2 (2.9)	6 (6.7)	0.273

Stroke	2 (1.2)	1 (1.4)	1 (1.1)	0.858

Ischemic heart disease	1 (0.6)	1 (1.4)	0 (0.0)	0.255

Kidney disease	1 (0.6)	1 (1.4)	0 (0.0)	0.255

Thyroid disease	1 (0.6)	0 (0.0)	1 (1.1)	0.376

On oral anticoagulation	21 (13.1)	13 (34.2)	6 (15.8)	0.064

Implantable cardioverter defibrillator	0	0	0	0

**Family and social history—*n* (%)**				

Smoking history	18 (11.2)	8 (11.4)	10 (11.1)	0.95

Alcohol use	43 (26.6)	13 (18.6)	30 (33.3)	0.037

Indoor air pollution	73 (45.6)	37 (52.9)	36 (40.0)	0.105

Family history of sudden cardiac death	1 (0.6)	1 (1.4)	0 (0.0)	0.255

**Nutrition status—*n* (%)**				0.247

Underweight	35 (23.3)	20 (31.2)	15 (17.4)	

Normal	76 (50.7)	30 (46.9)	46 (53.5)	

Overweight	29 (19.3)	10 (15.6)	19 (22.1)	

Obese	10 (6.7)	4 (6.2)	6 (7.0)	

**Vital signs at admission—median (IQR)**				

SBP	110 (90, 120)	100 (90, 120)	110 (92, 130)	0.127

DBP	69.5 (60, 80)	60 (60, 80)	70 (60, 80)	0.071

Heart rate	102 (85, 121)	113 (89.7, 138.2)	95.5 (84, 110.7)	0.002

Temperature	36.3(36, 36.5)	36.2 (36, 36.6)	36.3 (36.1, 36.5)	0.791

Respiratory rate	23 (20, 28)	24 (20, 28.7)	22 (20, 27)	0.293

SPO2	94 (91, 96)	95 (91, 97.2)	93 (92, 96)	0.083

**Baseline lab parameters—mean (*SD*)**				

Serum creatinine—umol/L	128 (100.7)	133 (100)	124 (101.6)	0.574

Urea—mmol/L	11.6 (9.74)	12.7 (10.9)	10.7 (8.7)	0.209

WBC—10^9/L^	9.9 (14.97)	11.7 (22.1)	8.5 (4.9)	0.199

Hemoglobin—g/dL	13.3 (10.63)	14.5 (15.9)	12.4 (2.5)	0.222

Platelets—10^9/L^	228 (100.3)	217 (104.1)	236 (97.1)	0.249

Potassium—mmol/L	4.7 (1.07)	4.7 (1.1)	4.6 (1)	0.534

Sodium—mmol/L	131 (11.7)	131 (9.2)	131 (13.4)	0.843

Albumin (g/L)	32 (6.6)	33 (6.7)	31.7 (6.5)	0.178

RBS—mmol/L	6.6 (3.42)	6.7 (3.7)	6.6 (3.2)	0.912

TSH—uMU	3.7 (4.88)	4.6 (6.5)	3.2 (3.6)	0.328

**COVID-19 Positive—*n* (%)**	16 (10)	3 (4.3)	13 (14.4)	0.1

**Requiring vasoactive medicine at admission—*n* (%)**	50 (31.2)	27 (38.6)	23 (25.6)	0.078

Norepinephrine	30 (18.8)	22 (31.4)	9 (10.0)	

Dobutamine	19 (11.9)	17 (24.2)	21 (23.3)	

Milrinone	1 (0.6)	0 (0)	1 (0.01)	

**Left ventricular ejection function—*n* (%)**				

≥50%	80 (50)	34 (48.6)	46 (51.1)	0.153

>40–<50%	29 (19.0)	17 (24.3)	12 (13.3)	

≤40%	44 (28.8)	18 (25.7)	26 (28.9)	

Not available (NA)	7 (4.4)	1 (1.4)	6 (6.7)	

**Diastolic dysfunction—*n* (%)**	27 (17.8)	7 (10.3)	20 (23.8)	0.03

**Right ventricular function**				

Normal	73 (47.7)	27 (39.1)	46 (54.8)	0.13

Mildly reduced	12 (7.8)	6 (8.7)	6 (7.1)	

Moderately reduced	22 (14.4)	9 (13.0)	13 (15.5)	

Severely reduced	46 (30.1)	27 (39.1)	19 (22.6)	


**Table 2:** Admission diagnosis.


ADMISSION DIAGNOSIS—*N* (%)	ARRHYTHMIA	

	YES (*N* = 70)	NO (*N* = 90)

Rheumatic heart disease (RHD)	35 (50)	11 (12.2)

Ischemic cardiomyopathy (IHD)	2 (2.9)	10 (11.1)

Cor pulmonale	3 (4.3)	12 (13.3)

Other cardiomyopathy	9 (12.9)	4 (4.4)

Pericardial disease	1 (1.4)	6 (6.7)

Congenital heart disease	3 (4.3)	1 (1.1)

Uncharacterized dilated cardiomyopathy	6 (8.6)	12 (13.3)

Hypertensive heart disease	6 (8.6)	10 (11.1)

Alcoholic dilated cardiomyopathy	1 (1.4)	7 (7.8)

Peripartum cardiomyopathy	1 (1.4)	6 (6.7)

Pulmonary embolism	0	5 (5.6)


**Figure 2:**
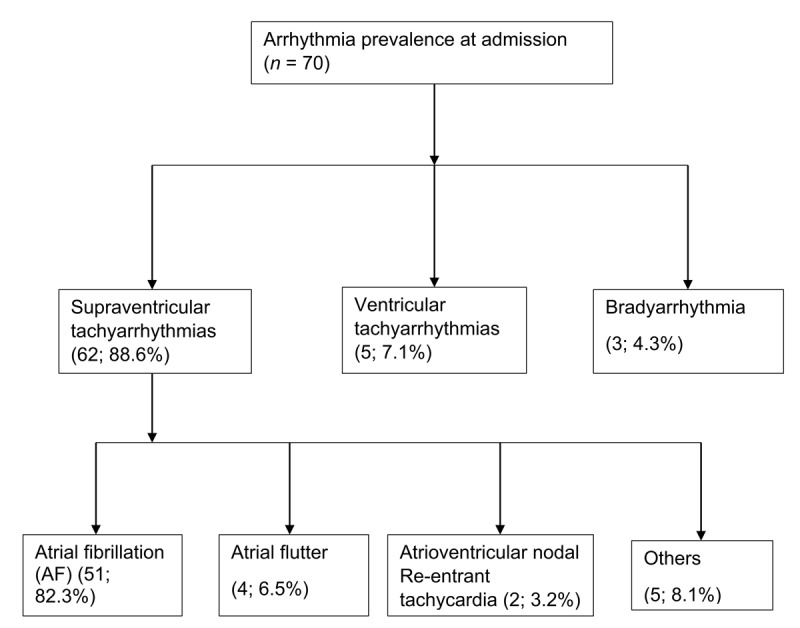
Types and proportion of cardiac arrhythmias.

Among the patients with arrhythmia, 12 (17.1%) were hemodynamically unstable. The most common arrhythmia subtype was supraventricular tachycardia in 62 (88.6%) of the patients, mainly AF (82.3%). Five (7.1%) had ventricular tachyarrhythmias, and three (4.3%) had bradyarrhythmia. The different subtypes of arrhythmia identified are summarized in Figure 2. Of the patients with arrhythmias, 22 (31%) underwent electrical cardioversion/defibrillation and 35 (50%) of the patients receiving pharmacological therapy were managed with digoxin. At discharge, 13 (18.6%) of patients admitted with an arrhythmia were in sinus rhythm. During follow-up, new onset arrhythmia (supraventricular arrhythmia) developed among three patients in the no arrhythmia group (Table 3).

**Table 3:** Management among patients with arrhythmias and incidence of arrhythmias in CCU.


PATIENT MANAGEMENT	FREQUENCY (%)

**Electrical (synchronized/defibrillation)—*n* (%)**	**22 (31)**

**Medical therapy—*n* (%)**	

Digoxin	35 (50)

Amiodarone	13 (18.6)

Metoprolol	4 (5.7)

**Pacing—*n* (%)**	**1 (1.4)**

**Resolution of arrhythmias at discharge—*n* (%)**	**13 (18.6)**

**New onset arrhythmia in CCU—*n* (%)**	

Supraventricular tachyarrhythmias	3 (0.03)

Bradyarrhythmia	0 (0)

Ventricular tachyarrhythmias	0 (0)


There was no significant difference in 30-day mortality among those with arrhythmia (23; 32.9%) compared to those without arrhythmia (27; 30.0%). The median length of hospital stay was higher in those without arrhythmia compared to those with arrhythmia [median = 9 (IQR = 6,14) vs median = 8 (IQR = 4,12), *p* value = 0.044]. The 30-day all-cause readmission was four (8.2%) in the arrhythmia group and one (1.5%) in the no arrhythmia group (*p* value = 0.087) (Table 4).

**Table 4:** 30-day outcomes among the patients.


OUTCOME	ARRHYTHMIA (*N* = 70)	NO ARRHYTHMIA (*N* = 90)	*P* VALUE

**Survival to 30 days—*n* (%)**			0.638

Alive	47 (67.1)	62 (68.9)	

Dead	23 (32.9)	27 (30.0)	

Unknown	0	1 (1.1)	

**Survival to discharge—*n* (%)**			0.821

Alive	51 (72.7)	67 (74.4)	

Dead	19 (27.1)	23 (25.6)	

**Length of stay—median (IQR**)	8 (4,12)	9 (6,14)	0.044

**30-day all-cause readmission—*n* (%)**	4 (8.2)	1 (1.5)	0.087


**Figure 3:**
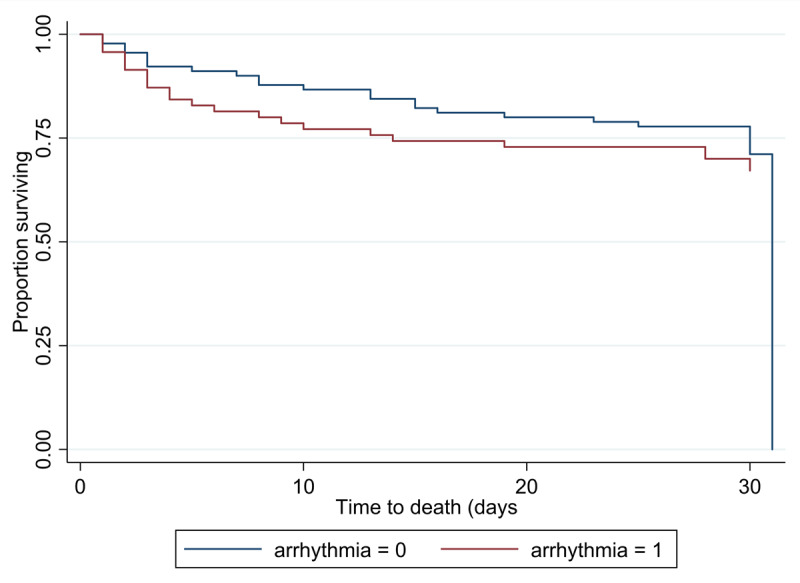
Kaplan-Meier curve showing overall 30 days survival by arrhythmia status at admission.

The difference in 30 days mortality, and readmission between the arrhythmia group and the no arrhythmia group, was also not significant after stratification by gender. There was higher proportion of deaths among male patients with arrhythmias (Table 5). Overall mortality did not significantly differ between the two groups and most deaths occurred within 10 days (Figure 3). On multivariate analysis, the risk of mortality among those with LVEF 40%–50% was less compared to those with LVEF of >50% (HR = 0.36, 95% CI 0.13, 0.99: *p* value = 0.049). In addition, the mortality risk among those on vasoactive meds was greater than that of patients not on vasoactive meds (HR = 2.9, 95% CI 1.5, 5.8), *p* value = 0.002 (Table 6).

**Table 5:** 30-day outcome stratified by gender among the participants.


VARIABLE	MALE			FEMALE		
	
	ARRHYTHMIA	NO ARRHYTHMIA	*P* VALUE	ARRHYTHMIA	NO ARRHYTHMIA	*P* VALUE
	
	*N* = 22	*N* = 43		*N* = 48	*N* = 47	

**Survival to discharge—*n*(%)**			0.232			0.609

**Alive**	15 (68.2)	36 (83.7)		35 (72.9)	32 (68.1)	

**Dead**	7 (31.8)	7 (16.3)		13 (27.1)	15 (31. 9)	

**Survival to 30 days—*n* (%)**						0.474

Alive	15 (68.2)	34 (79.1)		32 (66.7)	28 (59.6)	

Dead	7 (31.8)	8 (18.6)		16 (33.3)	19 (40.4)	

Unknown	0 (0)	1 (2.3)		0 (0)	0 (0)	

**Length of stay—median (IQR)**			0.819			0.04

	8.5 (7.25, 16.75)	11 (5.3, 17.5)		7 (4,10)	9 (6.1, 13.25)	

**30-day all-cause readmission—*n* (%)**			0.123			0.365

Yes	1 (6.7)	0 (0)		3 (8.8)	1 (3.3)	


**Table 6:** Multivariate analysis of factors associated with 30-day mortality.


VARIABLE	HAZARD RATIO	*P* VALUE	LOWER LIMIT[95% CONF.	UPPER LIMITINTERVAL]

Arrhythmia (yes vs no)	1.096	0.813	0.513	2.344

Age	1.001	0.883	0.984	1.019

Gender (female vs male)	1.496	0.294	0.705	3.176

Heart failure present (yes vs no)	0.775	0.688	0.223	2.69

NYHA (3,4 vs 1,2)	1.844	0.174	0.763	4.455

RHD present (yes vs no)	1.118	0.843	0.371	3.368

HTN present (yes vs no)	0.568	0.31	0.191	1.692

History of arrhythmia (yes vs no)	0.844	0.741	0.308	2.308

LVEF >40–<50% vs >50%	0.358	**0.049**	0.129	0.997

LVEF ≤40% vs >50%	0.463	0.137	0.168	1.277

LVEF NA vs >50%	0.864	0.826	0.236	3.169

Valvular (NA vs mild/normal)	1.550	0.346	0.622	3.860

Valvular (moderate/severe vs mild/normal)	1.548	0.314	0.661	3.627

Vasoactive meds (yes vs no)	2.924	**0.002**	1.486	5.754

RV function (moderate/severe vs mild/normal)	1.431	0.352	0.672	3.046

Creatinine (abnormal vs normal)	1.472	0.314	0.694	3.123

Urea (abnormal vs normal)	1.943	0.085	0.912	4.138

Sodium (abnormal vs normal)	0.865	0.693	0.42	1.781

Albumin (abnormal vs normal)	0.59	0.123	0.301	1.153


## Discussion

In a public sector CCU in Western Kenya, we detected a relatively high prevalence of arrhythmia upon admission to the CCU. Supraventricular tachyarrhythmias were the most common, with AF constituting the majority. In contrast to our a priori hypothesis, the primary outcome of all-cause mortality was similar between the patients who were admitted with a diagnosis of arrhythmia and those without. However, despite being relatively young, both groups had a high 30-day mortality rate of 30% or more. To the best of our knowledge, this is the first study looking at a population of CCU patients in Africa with a fairly large sample size and good follow-up.

The high prevalence of arrhythmia reflects the structurally distinct cardiac pathologies in SSA, where RHD is still an enormous burden, being more prevalent in younger and female patients [14,16,27,28]. In contrast, studies from HICs report a lower prevalence of arrhythmia. For example, in two multicenter studies, the prevalence was 10% in the Tokyo CCU registry and 17.1% in the North American study [4,29]. A recent systematic review reported a rising prevalence of arrhythmia in SSA [30]. This may reflect either a true rising prevalence or improved diagnostic capacity. For example, the Pan African Society of Cardiology (PASCAR) initiatives have led to improved training of personnel and pacing activities in SSA [17]. Nevertheless, our findings indicate the need for screening for arrhythmias in patients with a critical illness.

Supraventricular tachyarrhythmias were the most common (88.6%) among patients with arrhythmia, with AF representing the majority at 82.3%. Similar to our cohort, a multicenter study of patients with AF in Africa and the Middle East showed it mainly affected young females [31]. In THESUS-HF study, the prevalence of AF among hospitalized heart failure patients was lower and varied from 7.7% to 18.3% [32]. However, their population was older and gender-balanced compared to this study. None of the studies in the African region were conducted in cohorts of critically ill patients. Compared with HICs whose population is older, our study revealed a high burden of AF [2,4]. Various reasons might account for the higher prevalence of AF in SSA. First, the majority of our study cohort with AF had RHD, which is still quite prevalent in Western Kenya [14,16]. Second, there is high prevalence of permanent AF in SSA, which is likely related to not only the tendency of clinicians to opt for rate control strategies even in cases of new onset AF [25,26,33], but also the lack of access to catheter ablation. Only 18.6% of all patients with arrhythmias were discharged in sinus rhythm. Additionally, paroxysmal AF has been shown to progress to permanent AF [45,46,47], and it is likely that underdiagnosis and/or inadequate management in SSA can lead to higher rate of progression to permanent AF. Finally, aging and urbanization are associated with rising cases of comorbidities associated with AF [34]. While there is reported higher prevalence of supraventricular tachyarrhythmias in HICs, data in SSA are lacking [30].

Ventricular tachyarrhythmias comprised 7.1% of those who had a diagnosis of arrhythmia. We found a lower prevalence of ventricular tachyarrhythmias than the studies from HICs; among those who had arrhythmia, the prevalence of ventricular tachyarrhythmias was 25% in the Tokyo registry and 16.9% in Puerto Rico [1,3,4]. In SSA, we found no studies reporting the prevalence of ventricular arrhythmias; however, few studies have reported the incidence of SCD which can occur after ventricular arrhythmia [24,35,36]. A population-based survey of SCD in Cameroon found an incidence rate of 24.2/100,000 person year [37]. Hence, the lower prevalence in SSA may be due to underdiagnosis leading to sudden deaths in the community, which are likely underreported and under investigated, or a lower burden of ischemic heart disease [20,35]. illustrative of this, none of our patients had an implanted ICD prior or during their hospital stay.

Bradyarrhythmia was the least common of all arrhythmia, constituting only 4.3% which was mostly due to AV block and was lower than 27% prevalence in the Tokyo registry [4]. Although the prevalence of bradyarrhythmia in Africa is unknown [6], previous studies have found that AV block is the most common indication for pacing in SSA [15,19,38]. Cardiac conduction abnormalities can cause heart failure and SCD resulting from extreme bradycardia [7]. The PASCAR survey established inadequate diagnostic and treatment services for arrhythmias in SSA [23]. Hence, underdiagnosis and SCD prior to pacing may explain the lower prevalence in this study.

The presence of an arrhythmia at admission had no significant impact on the primary outcome of 30-day all-cause mortality, in contrast to those from HICs, which have reported higher mortality among patients with arrhythmias [3,39,40,41]. Our findings may reflect prompt diagnosis and better management for patients with arrhythmias given that it is an academic center with a highly trained dedicated staff establishment. We also postulate that differences in the common underlying heart disease between the two groups may be in play. Cor pulmonale and cardiomyopathies were more common in the no arrhythmia group. Cor pulmonale tends to be more common in the elderly, while cardiomyopathies are associated with poorer outcomes [32,42]. Further, there were more males in the no arrhythmia group who on subgroup analysis appeared to do worse in terms of outcomes. In addition, the study was also conducted during the COVID-19 pandemic, a period that saw patients delay presentation to the hospital which might have increased their overall mortality risk. On multivariate analysis, the use of vasoactive agents was significantly associated with increased 30-day mortality, similar to prior studies [29,39].

Although the 30-day duration of follow-up was short, many similar studies have used a similar period [3,39,43], thus our results may be best comparable to these studies. Sub-analysis of the outcomes based on arrhythmia subtypes was not feasible given the small number of the ventricular arrhythmias and bradyarrhythmia. We also had a very low incidence of tachyarrhythmias in our study. Hence, despite the above observations on overall mortality, the short duration of follow-up and the small sample size preclude firm conclusions but build a foundation for our future studies.

Of the secondary outcomes, we found a significant yet marginal increase in the length of stay among patients without arrhythmia of one day. This finding contrasted a systematic review that identified arrhythmia as one of the factors associated with increased length of stay in the ICU [44]. Notably, in addition to having a higher proportion of cor pulmonale and cardiomyopathies as mentioned earlier, more patients in the no arrhythmia group were diagnosed with COVID-19, which may have impacted their length of stay.

### Study Limitations

We acknowledge that our study is not without limitations. First, this study was done in a specialized cardiac care unit in a tertiary referral center which limits its generalizability to other settings. Second, this being an observational study, we cannot fully control confounders and patients were not matched at enrollment. Third, the mortality in our study in the no arrhythmia group was higher than the hypothesized value, which might have lowered the power to detect the mortality difference between the two groups. Fourth, arrhythmias were not classified as new or preexisting or analyzed by the subtypes due to the small numbers, which may have led to different conclusions. Fifth, paroxysmal arrhythmias may have been misclassified since history of arrhythmia was not factored in the patient assignments. Finally, the high prevalence of AF in our population limits the extrapolation of our findings to populations with different arrhythmia prevalence.

## Conclusions

Supraventricular tachyarrhythmias were common in critically hospitalized cardiac patients in Western Kenya, with AF constituting the majority. Thirty-day all-cause mortality was moderately high and did not differ between the group admitted with a diagnosis of arrhythmia and those without. Future multicenter studies with larger sample sizes, baseline matching and longer follow-up are required in Africa to further characterize the impact of arrhythmia in critically ill patients with cardiac disease.

## References

[1] Valderrábano RJ, Blanco A, Santiago-Rodriguez EJ, et al. Risk factors and clinical outcomes of arrhythmias in the medical intensive care unit. Journal Intensive Care. 2016; 4: 9. DOI: 10.1186/s40560-016-0131-x PMC4724077 26807261

[2] Tongyoo S, Permpikul C, Haemin R, Epichath N. Predicting factors, incidence and prognosis of cardiac arrhythmia in medical, non-acute coronary syndrome, critically ill patients. Journal of the Medical Association of Thailand. 2013; 96(Suppl 2): S238–45. https://europepmc.org/article/med/23590048. 23590048

[3] Annane D, Sébille V, Duboc D, et al. Incidence and prognosis of sustained arrhythmias in critically ill patients. American Journal of Respiratory and Critical Care Medicine. 2008; 178(1): 20–25. DOI: 10.1164/rccm.200701-031OC 18388358

[4] Kobayashi Y. How to manage various arrhythmias and sudden cardiac death in the cardiovascular intensive care. Journal of Intensive Care. 2018; 6(1): 23. DOI: 10.1186/s40560-018-0292-x 29686877 PMC5896158

[5] Papadopoulos CH, Oikonomidis D, Lazaris E, Nihoyannopoulos P. Echocardiography and cardiac arrhythmias. Hellenic Journal of Cardiology. 2018; 59(3): 140–149. DOI: 10.1016/j.hjc.2017.11.017 29203161

[6] Adedinsewo D, Omole O, Oluleye O, Ajuyah I, Kusumoto F. Arrhythmia care in Africa. Journal of Interventional Cardiac Electrophysiology. 2019; 56: 127–135. DOI: 10.1007/s10840-018-0398-z 29931543

[7] Arnar DO, Raatikainen MJ. Promoting cardiac arrhythmia care in Africa: A big challenge that begins with data. EP Europace. 2018; 20(9): 1397–1398. DOI: 10.1093/europace/eux352 29309583

[8] Bonny A, Ngantcha M, Jeilan M, et al. Statistics on the use of cardiac electronic devices and interventional electrophysiological procedures in Africa from 2011 to 2016: Report of the Pan African Society of Cardiology (PASCAR) Cardiac Arrhythmias and Pacing Task Forces. EP Europace. 2017; 20(9): 1513–1526. DOI: 10.1093/europace/eux353 PMC6123943 29309556

[9] Kloosterman M, Conen D, Oldgren J, et al. 47 Characteristics and outcomes of atrial fibrillation in patients without conventional risk factors: A RE-LY AF registry analysis. EP Europace. 2018; 20(suppl_1). DOI: 10.1093/europace/euy015.009 PMC7273333 32215649

[10] Bonny A, Ngantcha M, Yuyun M, et al. Cardiac arrhythmia services in Africa from 2011 to 2018: The second report from the Pan African Society of Cardiology working group on cardiac arrhythmias and pacing. EP Europace. 2020; 22(3): 420–433. DOI: 10.1093/europace/euz354 31989158

[11] Chugh SS, Havmoeller R, Narayanan K, et al. Worldwide epidemiology of atrial fibrillation: A Global Burden of Disease 2010 Study. Circulation. 2014; 129(8): 837–847. DOI: 10.1161/CIRCULATIONAHA.113.005119 24345399 PMC4151302

[12] Morillo CA, Banerjee A, Perel P, Wood D, Jouven X. Atrial fibrillation: The current epidemic. Journal of Geriatric Cardiology. 2017; 14(3): 195–203. DOI: 10.11909/j.issn.1671-5411.2017.03.011 28592963 PMC5460066

[13] Bosch NA, Cimini J, Walkey AJ. Atrial fibrillation in the ICU. Chest. 2018; 154(6): 1424–1434. DOI: 10.1016/j.chest.2018.03.040 29627355 PMC6335260

[14] Oldgren J, Healey JS, Ezekowitz M, et al. Variations in cause and management of atrial fibrillation in a prospective registry of 15 400 emergency department patients in 46 countries: The RE-LY atrial fibrillation registry. Circulation. 2014; 129(15): 1568–1576. DOI: 10.1161/CIRCULATIONAHA.113.005451 24463370

[15] Adoubi K, Kane AD, Coulibaly I, et al. The atrial fibrillation registry in countries of Africa: Rationale and design of Africa. Archives of Cardiovascular Diseases Supplements. 2018; 10(1): 87. DOI: 10.1016/j.acvdsp.2017.11.234

[16] Temu TM, Lane KA, Shen C, et al. Clinical characteristics and 12-month outcomes of patients with valvular and non-valvular atrial fibrillation in Kenya. PLoS One. 2017; 12(9): e0185204. DOI: 10.1371/journal.pone.0185204 28934312 PMC5608343

[17] Kane A, Sarr SA, Ndobo JVD, et al. Cardiac pacing challenge in Sub-Saharan Africa environment: Experience of the Cardiology Department of Teaching Hospital Aristide Le Dantec in Dakar. BMC Cardiovascular Disorders. 2019; 19(1): 1–7. DOI: 10.1186/s12872-019-1176-2 31412773 PMC6694489

[18] Falase B, Sanusi M, Johnson A. Analysis of a five-year experience of permanent pacemaker implantation at a Nigerian teaching hospital: Need for a national database. Pan African Medical Journal. 2014; 16(16): 16. DOI: 10.11604/pamj.2013.16.16.2644 PMC3909697 24498465

[19] Thomas MO, Oke DA, Ogunleye EO, Adeyanju FA. Bradypacing: Indications and management challenges in Nigeria. Pacing and Clinical Electrophysiology. 2007; 30(6): 761–763. DOI: 10.1111/j.1540-8159.2007.00747.x 17547609

[20] Bonny A, Ngantcha M, Scholtz W, et al. Cardiac arrhythmias in Africa: Epidemiology, management challenges, and perspectives. Journal of the American College of Cardiology. 2019; 73(1): 100–109. DOI: 10.1016/j.jacc.2018.09.084 30621939

[21] Wong CX, Brown A, Lau DH, et al. Epidemiology of sudden cardiac death: Global and regional perspectives. Heart Lung and Circulation. 2019; 28(1): 6–14. DOI: 10.1016/j.hlc.2018.08.026 30482683

[22] Chin A. Sudden cardiac death in Africa. Cardiovascular Journal of Africa. 2014; 25(4): 151. 25192296 PMC4170171

[23] Talle MA, Bonny A, Scholtz W, et al. Status of cardiac arrhythmia services in Africa in 2018: A PASCAR Sudden Cardiac Death Task Force report. Cardiovascular Journal of Africa. 2018; 29(2): 115. DOI: 10.5830/CVJA-2018-027 29745966 PMC6008897

[24] Vedanthan R, Fuster V, Fischer A. Sudden cardiac death in low and middle-income countries. Global Heart. 2012; 7(4): 353–360. DOI: 10.1016/j.gheart.2012.10.002 25689944 PMC4363741

[25] Stambler BS, Ngunga LM. Atrial fibrillation in Sub-Saharan Africa: Epidemiology, unmet needs, and treatment options. International Journal of General Medicine. 2015; 8: 231. DOI: 10.2147/IJGM.S84537 26261423 PMC4527570

[26] Jardine RM, Fine J, Obel IWP. A survey on the treatment of atrial fibrillation in South Africa. South African Medical Journal. 2014; 104(9): 623–627. DOI: 10.7196/SAMJ.8111 25212404

[27] Zühlke L, Engel ME, Karthikeyan G, et al. Characteristics, complications, and gaps in evidence-based interventions in rheumatic heart disease: The Global Rheumatic Heart Disease Registry (the REMEDY study). European Heart Journal. 2014; 36(18): 1115–1122. DOI: 10.1093/eurheartj/ehu449 25425448 PMC4422972

[28] Sliwa K, Davison BA, Mayosi BM, et al. Readmission and death after an acute heart failure event: Predictors and outcomes in sub-Saharan Africa: Results from the THESUS-HF registry. European Heart Journal. 2013; 34(40): 3151–3159. DOI: 10.1093/eurheartj/eht393 24048728

[29] Bohula EA, Katz JN, van Diepen S, et al. Demographics, care patterns, and outcomes of patients admitted to cardiac intensive care units: The Critical Care Cardiology Trials Network Prospective North American Multicenter Registry of Cardiac Critical Illness. JAMA Cardiology. 2019; 4(9): 928–935. DOI: 10.1001/jamacardio.2019.2467 31339509 PMC6659157

[30] Yuyun MF, Bonny A, Ng GA, et al. A systematic review of the spectrum of cardiac arrhythmias in sub-Saharan Africa. Global Heart. 2020; 15(1): 37. DOI: 10.5334/gh.808 32923331 PMC7413135

[31] Gamra H, Murin J, Chiang CE, Naditch-Brûlé L, Brette S, Steg PG. Use of antithrombotics in atrial fibrillation in Africa, Europe, Asia and South America: Insights from the International RealiseAF Survey. Archives of Cardiovascular Disease. 2014; 107(2): 77–87. DOI: 10.1016/j.acvd.2014.01.001 24556189

[32] Damasceno A, Mayosi BM, Sani M, et al. The causes, treatment, and outcome of acute heart failure in 1006 Africans from 9 countries: Results of the sub-Saharan Africa survey of heart failure. Archives of Internal Medicine. 2012; 172(18): 1386–1394. https://jamanetwork.com/journals/jamainternalmedicine/articlepdf/1356531/ioi120046_1386_1394.pdf (accessed 25 June 2022). DOI: 10.1001/archinternmed.2012.3310 22945249

[33] Ntep-Gweth M, Zimmermann M, Meiltz A, et al. Atrial fibrillation in Africa: Clinical characteristics, prognosis, and adherence to guidelines in Cameroon. EP Europace. 2010; 12(4): 482–487. https://www.mendeley.com/catalogue/c9eeb5df-e7e8-3f36-ba04-97ae35875ced (accessed 24 June 2022). DOI: 10.1093/europace/euq006 20179174

[34] Shavadia J, Yonga G, Mwanzi S, Jinah A, Moriasi A, Otieno H. Clinical characteristics and outcomes of atrial fibrillation and flutter at the Aga Khan University Hospital, Nairobi. Cardiovascular Journal of Africa. 2013; 24(2): 6–9. DOI: 10.5830/CVJA-2012-064 23612946 PMC3734872

[35] Bonny A, Amougou SN, Noah DN, Mbenoun ML, Karaye K. Sudden cardiac death in low-resource settings: Lessons from a resuscitated cardiac arrest. Cardiovascular Journal of Africa. 2015; 26(2): 91–95. DOI: 10.5830/CVJA-2015-020 25940123

[36] Ogunlade O. Sudden cardiac death in Nigeria: A health challenge. International Journal of Health Research. 2011; 4(4): 163–168. https://www.researchgate.net/profile/Oluwadare-Ogunlade/publication/266461340_Sudden_Cardiac_Death_in_Nigeria_A_Health_Challenge/links/54b409790cf26833efcff07c/Sudden-Cardiac-Death-in-Nigeria-A-Health-Challenge.pdf.

[37] Bonny A, Tibazarwa K, Mbouh S, et al. Epidemiology of sudden cardiac death in Cameroon: The first population-based cohort survey in sub-Saharan Africa. International Journal of Epidemiology. 2017; 46(4): 1230–1238. DOI: 10.1093/ije/dyx043 28453817 PMC5837681

[38] dos Santos LA, Agathangelou NE, Taams MA, Lewis BS. Permanent cardiac pacing in South African blacks. South African Medical Journal. 1982; 61(25): 947–949. https://journals.co.za/doi/pdf/10.10520/AJA20785135_14706. 7089761

[39] Duarte PAD, Leichtweis GE, Andriolo L, et al. Factors associated with the incidence and severity of new-onset atrial fibrillation in adult critically ill patients. Critical Care Research and Practice. 2017; 2017: 8046240. DOI: 10.1155/2017/8046240 28702263 PMC5494087

[40] Reinelt P, Karth GD, Geppert A, Heinz G. Incidence and type of cardiac arrhythmias in critically ill patients: A single center experience in a medical-cardiological ICU. Intensive Care Medicine. 2001; 27(9): 1466–1473. DOI: 10.1007/s001340101043 11685339

[41] Artucio H, Pereira M. Cardiac arrhythmias in critically ill patients: Epidemiologic study. Critical Care Medicine. 1990; 18(12): 1383–1388. DOI: 10.1097/00003246-199012000-00015 2245612

[42] Lagat DK, Carter EJ, Kimaiyo S, Velazques E, Sherman CB. The Relationship of indoor air pollution (IAP) exposure to isolated right heart failure (IRHF) in women of Western Kenya: A pilot study. American Journal of Respiratory and Critical Care Medicine. 2012; 185: A1753. DOI: 10.1164/ajrccm-conference.2012.185.1_MeetingAbstracts.A1753

[43] Kang HM, Ng SJ, Yap S, Annathurai A, Ong ME. Outcomes of patients presenting with primary or secondary atrial fibrillation with rapid ventricular rate to the emergency department. Annals of the Academy of Medicine Singapore. 2018; 47(11): 438–444. DOI: 10.47102/annals-acadmedsg.V47N11p438 30578422

[44] Almashrafi A, Elmontsri M, Aylin P. Systematic review of factors influencing length of stay in ICU after adult cardiac surgery. BMC Health Services Research. 2016; 16: 318. DOI: 10.1186/s12913-016-1591-3 27473872 PMC4966741

[45] Proietti R, Hadjis A, AlTurki A, Thanassoulis G, Roux JF, Verma A, … Essebag V. A systematic review on the progression of paroxysmal to persistent atrial fibrillation: shedding new light on the effects of catheter ablation. JACC: Clinical Electrophysiology. 2015; 1(3): 105–115. DOI: 10.1016/j.jacep.2015.04.010 29759352

[46] Padfield GJ, Steinberg C, Swampillai J, Qian H, Connolly SJ, Dorian P, … Kerr CR. Progression of paroxysmal to persistent atrial fibrillation: 10-year follow-up in the Canadian Registry of Atrial Fibrillation. Heart rhythm. 2017; 14(6): 801–807. DOI: 10.1016/j.hrthm.2017.01.038 28232263

[47] De Vos CB, Pisters R, Nieuwlaat R, Prins MH, Tieleman RG, Coelen RJS, … Crijns HJ. Progression from paroxysmal to persistent atrial fibrillation: clinical correlates and prognosis. Journal of the American College of Cardiology. 2010; 55(8): 725–731. DOI: 10.1016/j.jacc.2009.11.040 20170808

